# Supplementary material for: Oral step-down vs full-course intravenous antibiotic therapy for infective endocarditis: Protocol for a systematic review and meta-analysis
Source: PLoS One. 2026 Apr 29;21(4):e0348084. doi: 10.1371/journal.pone.0348084 (PMC13127948; doi:10.1371/journal.pone.0348084)
Supplement: S1 Table — (DOCX) [file pone.0348084.s001.docx]

**S1 Table**. **Checklist PRISMA-P 2015 Checklist**

| **Section and topic** | **Item No** | **Checklist item** | **Information reported** | **Page/Section in protocol** |
| --- | --- | --- | --- | --- |
| **ADMINISTRATIVE INFORMATION** |  |  |  |  |
| Title: Identification | 1a | Identify the report as a protocol of a systematic review | Yes | Title page / Abstract |
| Title: Update | 1b | If protocol is an update | No | – |
| Registration | 2 | Provide name of registry and number | Yes – PROSPERO  CRD420251104423 | Methods: Study design |
| Authors: Contact | 3a | Provide names, affiliations, email, address of corresponding author | Yes | Title page |
| Authors: Contributions | 3b | Describe contributions and guarantor | Yes | Author contributions section |
| Amendments | 4 | If protocol is an amendment, describe changes | Not applicable; | -- |
| Support: Sources | 5a | Sources of support | None reported | Administrative |
| Support: Sponsor | 5b | Name of funder/sponsor | None | – |
| Support: Role of sponsor | 5c | Role of sponsor/funder | None | – |
| **INTRODUCTION** |  |  |  |  |
| Rationale | 6 | Describe rationale for the review | Yes | Introduction |
| Objectives | 7 | Explicit statement of review question (PICO) | Yes | Introduction / Objectives |
| **METHODS** |  |  |  |  |
| Eligibility criteria | 8 | Study characteristics (PICO, design, timeframe, language) | Yes | Methods: Eligibility criteria |
| Information sources | 9 | All intended information sources, dates | Yes | Methods: Information sources |
| Search strategy | 10 | Draft search strategy | Yes (MEDLINE strategy provided) | Appendix / Methods |
| Study records: Data management | 11a | Mechanisms to manage records | Yes – Rayyan, REDCap | Methods: Screening, Data extraction |
| Study records: Selection process | 11b | Process for selecting studies | Yes – two reviewers, third adjudicator | Methods: Screening procedure |
| Study records: Data collection process | 11c | Planned data extraction, duplicate, contacting authors | Yes | Methods: Data extraction |
| Data items | 12 | Variables to be extracted | Yes – study, population, intervention, comparator, outcomes | Methods: Data extraction |
| Outcomes and prioritization | 13 | List of outcomes, prioritization | Yes – primary: mortality; secondary: relapse, surgery, embolic events, LOS, adverse events | Methods: Outcomes |
| Risk of bias in individual studies | 14 | Anticipated methods | Yes – RoB 2, ROBINS-I | Methods: Risk of bias |
| Data synthesis | 15a | Criteria for quantitative synthesis | Yes | Methods: Data synthesis |
|  | 15b | Planned summary measures, handling data, heterogeneity | Yes – RR, MD/SMD, I² | Methods: Data synthesis |
|  | 15c | Additional analyses | Yes – subgroup and sensitivity analyses | Methods: Data synthesis |
|  | 15d | If quantitative synthesis not appropriate | Narrative summary planned | Methods |
| Meta-bias(es) | 16 | Assessment of meta-biases | Yes – publication bias, funnel plots if ≥10 studies | Methods |
| Confidence in cumulative evidence | 17 | How evidence strength will be assessed | Yes – GRADE | Methods: Certainty of evidence |
