# Supplementary material for: Oral step-down vs full-course intravenous antibiotic therapy for infective endocarditis: Protocol for a systematic review and meta-analysis
Source: PLoS One. 2026 Apr 29;21(4):e0348084. doi: 10.1371/journal.pone.0348084 (PMC13127948; doi:10.1371/journal.pone.0348084)
Supplement: S2 Table — (DOCX) [file pone.0348084.s002.docx]

# **S2 Table. Search Strategy**

| **Database** | **Search equation** |
| --- | --- |
| Medline | ("PO antibiotics" OR "oral drug administration" OR "sequential therapy" OR "de-escalation" OR "switch therapy" OR "oral antibiotics" OR ("Administration, Oral"[MeSH] AND "Anti-Bacterial Agents"[MeSH]) OR "oral therapy" OR "step-down therapy") AND endocardit* |
| Embase (Elsevier) | #1 'endocarditis'/exp OR 'endo-carditis' OR 'endocardial inflammation' OR 'endocarditis' OR 'endocardium inflammation' OR 'paraneoplastic endocarditis' OR 'parietal fibroplastic endocarditis'  #2 'antiinfective agent'/exp OR 'anti bacterial agent' OR 'anti bacterial agents' OR 'anti infective agents' OR 'anti infectives, otic' OR 'anti-bacterial agents' OR 'anti-infective agents' OR 'anti-infectives, otic' OR 'antibacterial' OR 'antibacterial agent' OR 'antibacterial drug' OR 'antibacterial soap' OR 'antibacterial spectrum' OR 'antiinfective agent' OR 'antiinfectives, otic' OR 'antimicrobial' OR 'antimicrobial agent' OR 'antimicrobial compound' OR 'antimicrobial drug' OR 'antimicrobial factor' OR 'antiseptic' OR 'antiseptic agent' OR 'antiseptic cream' OR 'antiseptic foam' OR 'antiseptic soap' OR 'chemotherapeutic agent' OR 'chemotherapeutic drug' OR 'chemotherapeutica' OR 'microbiological agent'  #3 'oral drug administration'/exp OR 'administration, oral' OR 'drug administration, oral' OR 'oral administration' OR 'oral drug administration' OR 'oral drug intake' OR 'p.o. administration' OR 'p.o. dosage' OR 'p.o. dose' OR 'p.o. drug administration' OR 'p.o. drug intake' OR 'per os drug administration'  #4  #1 AND #2  #5  #3 AND #4 |
| CENTRAL | EBM Reviews - Cochrane Central Register of Controlled Trials  1 endocarditis.mp.  2 PO antibiotics.mp.  3 "oral drug administration".mp.  4 sequential therapy.mp.  5 de-escalation.mp.  6 switch therapy.mp.  7 oral antibiotics.mp.  8 oral therapy.mp.  9 "step-down therapy".mp.  10 Administration, Oral/  11 2 or 3 or 4 or 5 or 6 or 7 or 8 or 9 or 10  12 1 and 11  13 Anti-Bacterial Agents/  14 12 and 13 |
| LILACS | (decs.subject:"Endocarditis Bacteriana" OR "endocarditis infecciosa" OR "bacterial endocarditis" OR "subacute bacterial endocarditis")  AND  (decs.subject:"Agentes Antibacterianos" OR antibiotics OR "terapia antibiótica" OR "antibiotic therapy" OR "antimicrobial therapy")  AND  (decs.subject:"Administración Oral" OR "antibióticos orales" OR "oral antibiotic therapy" OR "oral therapy" OR "vía oral" OR "PO antibiotics" OR "oral drug administration" OR "step-down therapy" OR "switch therapy" OR "sequential therapy" OR "de-escalation") |
