# Supplementary material for: Oral step-down vs full-course intravenous antibiotic therapy for infective endocarditis: Protocol for a systematic review and meta-analysis
Source: PLoS One. 2026 Apr 29;21(4):e0348084. doi: 10.1371/journal.pone.0348084 (PMC13127948; doi:10.1371/journal.pone.0348084)
Supplement: Attachment — Submitted filename: Response to reviewers.docx [file pone.0348084.s004.docx]

**Response to Editor and Reviewers regarding:**

**PONE-D-25-59799**

Oral step-down vs full-course intravenous antibiotic therapy for infective endocarditis: Protocol for a systematic review and meta-analysis

PLOS One

Dear Editor/Reviewers,

We thank the Editor and the Reviewers for their careful evaluation of our protocol and for their constructive comments, which have helped us improve the clarity, rigor, and transparency of the manuscript. Below, we provide a detailed, point‑by‑point response. All suggested changes have been incorporated into the revised version of the manuscript, and the relevant sections are explicitly indicated.

**Journal Requirements**

**1. PLOS ONE formatting and file naming requirements**

We appreciate this reminder. The revised submission complies with all PLOS ONE style and formatting requirements. Specifically:

- The manuscript has been reformatted using the official PLOS ONE templates for the main body and title/authors/affiliations.
- File names have been updated to follow PLOS ONE conventions.
- Line numbering, section structure, reference formatting, table placement, and supporting information labels have been verified and corrected where needed.

**2. Citation of suggested literature**

No citations suggestions were made by reviewers

**Reviewer #1**

We thank Reviewer #1 for their positive and supportive assessment of the scientific rationale, methodological rigor, feasibility, and clarity of the protocol. No specific revisions were requested beyond those already addressed.

**Reviewer #2**

We thank Reviewer #2 for their thoughtful and detailed feedback. Each comment is addressed individually below.

- Comment 1: The rationale should be better elaborated (expected outcomes, patient categories that may benefit most from oral therapy).

**Response:**
We agree and have strengthened the rationale substantially. The Introduction has been expanded to clarify that the goal of oral step‑down therapy is non‑inferiority in clinical outcomes (efficacy), with potential secondary benefits including reduced catheter‑related complications, shorter hospital stays, lower costs, and improved quality of life.

We agree with the reviewer that infective endocarditis represents a highly complex and heterogeneous population, and that the clinical plausibility of oral step‑down therapy is likely greater in less complex scenarios. In the revised manuscript, we now explicitly acknowledge that oral transition may be more feasible in patients with native valve endocarditis, left‑sided disease, low‑virulence microorganisms, and absence of major complications or high‑risk features.

Importantly, we clarify that this represents a biologically and clinically informed hypothesis rather than a restriction of eligibility. To ensure a comprehensive and externally valid evidence synthesis, all types of infective endocarditis are included, and differences according to valve type, anatomical location, microbiology, study design, and clinical complexity will be explored through prespecified subgroup and sensitivity analyses.

- Comment 2: Specify the years of enrollment of included studies.

**Response:**

We acknowledge this important point. The Search Strategy and Eligibility Criteria sections now explicitly state the temporal coverage of the review. We will include studies published from database inception through January 2026, corresponding to the contemporary era of oral bioavailable antibiotic regimens and modern diagnostic criteria. We intentionally avoid restricting the review to an arbitrary start year, as earlier studies may still inform outcomes and heterogeneity, but we clarify the end date clearly and justify inclusivity.

- Comment 3: Clarify how heterogeneity will be managed (study design, retrospective vs prospective, native vs prosthetic valves, off‑label use).

**Response:**
We fully agree and have clarified our approach. The Data synthesis and statistical analysis section has been expanded to explicitly describe anticipated clinical and methodological heterogeneity. Pre‑specified subgroup analyses, including Randomized vs observational studies, Retrospective vs prospective designs, Native vs prosthetic valve endocarditis, Left‑ vs right‑sided IE, Microorganism‑specific analyses where feasible. Planned sensitivity analyses, excluding high‑risk‑of‑bias studies and off‑label treatment cohorts when appropriate. Use of random‑effects models and narrative synthesis when pooling is inappropriate.

- Comment 4: Discuss more clearly the added value of publishing a systematic review protocol.

**Response:**
We appreciate this comment and have addressed it directly. The Discussion now includes a dedicated paragraph explaining the specific added value of protocol publication. Publication of the planned methodology ensures methodological transparency and protection against selective outcome reporting, allows peer scrutiny before results are known, reduces duplication of ongoing reviews and enhances credibility in areas with high clinical controversy and heterogeneous evidence, such as oral step‑down therapy for infective endocarditis.

This publication aligns with best practices advocated by PRISMA‑P, PROSPERO, and PLOS ONE.

- Data Availability

We have clarified the data availability statement to explicitly state that this manuscript corresponds to a systematic review protocol and does not involve primary data collection. All analyzed data will derive from published studies, and extracted datasets and analytic materials will be made available upon reasonable and methodologically pertinent request to the corresponding author. We believe this addresses transparency requirements while remaining appropriate for a protocol-stage publication.

We believe that the revisions undertaken in response to the reviewers’ comments have substantially strengthened the scientific justification, methodological clarity, and transparency of this protocol. We respectfully submit the revised manuscript for reconsideration and remain grateful for the opportunity to improve our work.

On behalf of the authors,

**Jose D. Cruz-Cuevas, MD
Fundación Cardioinfantil – Instituto de Cardiología
Bogotá, Colombia**
[**jcruz@lacardio.org**](mailto:jcruz@lacardio.org)
